# Characterization of the endocannabinoid system in subcutaneous adipose tissue in periparturient dairy cows and its association to metabolic profiles

**DOI:** 10.1371/journal.pone.0205996

**Published:** 2018-11-07

**Authors:** Maya Zachut, Gitit Kra, Uzi Moallem, Lilya Livshitz, Yishai Levin, Shiran Udi, Alina Nemirovski, Joseph Tam

**Affiliations:** 1 Department of Ruminant Science, Institute of Animal Sciences, Volcani Center, Rishon Lezion, Israel; 2 The Nancy and Stephen Grand Israel National Center for Personalized Medicine, Weizmann Institute of Science, Rehovot, Israel; 3 Obesity and Metabolism Laboratory, Institute for Drug Research, School of Pharmacy, Faculty of Medicine, the Hebrew University of Jerusalem, Jerusalem, Israel; University of Illinois, UNITED STATES

## Abstract

Adipose tissue (AT) plays a major role in metabolic adaptations in postpartum (PP) dairy cows. The endocannabinoid (eCB) system is a key regulator of metabolism and energy homeostasis; however, information about this system in ruminants is scarce. Therefore, this work aimed to assess the eCB system in subcutaneous AT, and to determine its relation to the metabolic profile in peripartum cows. Biopsies of AT were performed at 14 d prepartum, and 4 and 30 d PP from 18 multiparous peripartum cows. Cows were categorized retrospectively according to those with high body weight (BW) loss (HWL, 8.5 ± 1.7% BW loss) or low body weight loss (LWL, 2.9 ± 2.5% BW loss) during the first month PP. The HWL had higher plasma non-esterified fatty acids and a lower insulin/glucagon ratio PP than did LWL. Two-fold elevated AT levels of the main eCBs, *N*-arachidonoylethanolamine (AEA) and 2-arachidonoylglycerol (2-AG), were found 4 d PP compared with prepartum in HWL, but not in LWL cows. AT levels of the eCB-like molecules oleoylethanolamide, palmitoylethanolamide, and of arachidonic acid were elevated PP compared with prepartum in all cows. The abundance of monoglyceride lipase (MGLL), the 2-AG degrading enzyme, was lower in HWL vs. LWL AT PP. The relative gene expression of the cannabinoid receptors *CNR1* and *CNR2* in AT tended to be higher in HWL vs. LWL PP. Proteomic analysis of AT showed an enrichment of the inflammatory pathways’ acute phase signaling and complement system in HWL vs. LWL cows PP. In summary, eCB levels in AT were elevated at the onset of lactation as part of the metabolic adaptations in PP dairy cows. Furthermore, activating the eCB system in AT is most likely associated with a metabolic response of greater BW loss, lipolysis, and AT inflammation in PP dairy cows.

## Introduction

In high-yielding dairy cows, adipose tissue (AT) plays an important role in the metabolic adaptations during the transition from late pregnancy to calving, and the onset of lactation. During pregnancy cows are in a lipogenic state, whereas as calving approaches a period of high energy demand for milk production results in a substantial mobilization of AT through lipolysis of lipid reserves [1,2]. There is considerable phenotypic variation among high-yielding dairy cows regarding the extent of energy mobilization in early lactation[3,4]. The variation in the metabolic response in PP dairy cows can be demonstrated by the degree of lipolysis during early lactation, which represents the energy deficit and the related metabolic adaptations in each cow. In a series of experimental studies, we defined the magnitude of the metabolic response in cows based on their extent of body weight loss during the first 5 weeks postpartum (PP), classifying them either as high weight loss (HWL) or low weight loss (LWL) cows[5–7]. Recently, we have shown that the degree of body weight loss PP is repetitive within cows between lactations, it represents an intrinsic trait in high-yielding dairy cows, and that it has long-term implications on their reproductive performance[7].

The endocannabinoid (eCB) system is a central regulator of metabolism and energy homeostasis in mammals[8–11]. The eCB system is involved in many physiological and pathological conditions; its role in metabolism and energy homeostasis in humans and in many murine models for obesity, insulin resistance, and glucose homeostasis, as well as fatty liver is well defined and documented [12–14]. The main eCBs, *N*-arachidonoylethanolamide (anandamide, AEA)[15] and 2-arachidonoylglycerol (2-AG)[16,17], are essential regulators of the rapid (non-genomic) and stress-related fine tuning of energy intake due to their fast and adaptive mechanisms of synthesis, release, and degradation[18–20]. The eCBs bind to the cannabinoid receptors, CB1 (*CNR1*) and CB2 (*CNR2)*, leading to modulation of intracellular processes [18]. The CB1 receptor, abundantly expressed throughout the central nervous system, regulates feeding and energy expenditure. It is also present in peripheral tissues that are important for metabolic homeostasis such as AT[14]. The CB2 receptor, mainly abundant within cells of the immune system, is a major modulator of immune function[21,22]. In adipocytes, the presence of the CB1 receptor has been demonstrated both in humans and in rodents[23–25], and enzymes for the production and degradation of eCBs have also detected in fat depots [26,27]. Generally, the biological actions of CB1 receptor activation in adipocytes are mainly associated with maximizing fatty acid *de novo* biosynthesis, triglyceride accumulation, and with minimizing lipolysis[14].

The role of the eCB system in the metabolic adaptation of dairy cows to the onset of lactation has been scarcely explored. Previously, increased hepatic expression of *CNR2* and monoglyceride lipase (*MGLL*), the main 2-AG degrading enzyme, in cows fed a moderate-energy diet, compared with those fed a low-energy diet at 7 d PP, was reported[28]. However, information on key elements of the eCB system in the AT of ruminants is lacking. Therefore, the objectives of the present study were to examine, for the first time, the presence of different elements of the eCB system in the AT of peripartum dairy cows, and to determine whether components of this system are changed in dairy cows that exhibit an altered metabolic response PP, as reflected in their degree of BW loss, their circulating metabolic profile, and the AT metabolic state, revealed by longitudinal proteomic analysis.

## Materials and methods

### Animals and experimental procedures

The experimental protocol for the study was approved by the Volcani Center Animal Care Committee (approval number IL 553/14) and the experiment was performed in accordance with relevant guidelines and regulations. The study was conducted at the Volcani Center experimental farm in Rishon Lezion, Israel. Eighteen high-yielding, 261 ± 5 d pregnant dry multiparous Israeli-Holstein dairy cows averaging 730 ± 68 kg of body weight (BW) and an average lactation number of 3.6 (range 2–6) participated in this study, conducted during the summer season (August–October). These cows were part of a large study that examined the effects of season on late pregnant cows[29]. The cows were not cooled during late pregnancy; however, they were exposed to five cooling sessions per day PP according to a routine cooling management at the dairy farm, as previously described[30]. Ambient temperature and relative humidity (RH) were recorded every 3 h by the Israel Meteorological Service (Bet Dagan, Israel). Maximum RH and minimum ambient temperature as well as thermal heat index (THI) calculations were determined[30]. All cows were group-housed in a covered pen with an adjacent outside yard, and fed *ad libitum* once a day at 1100 h with a standard Israeli diet. The typical Israeli diet contains relatively high concentrate (65–67%), and low forage (33–35%).The composition and nutritional value of the pre- and PP diets are presented in **Table 1**.

**Table 1:** Ingredients and chemical compositions of prepartum and postpartum diets.

Diet	Prepartum Postpartum	
Ingredients, % of dry matter		
Corn, ground	11.0	26.5
Barley, rolled	0.7	1.7
Wheat grain, rolled	0.7	1.7
Rapeseed meal	1.9	4.5
Soybean meal	1.5	3.5
Gluten feed	3.7	9.0
Cottonseed	0.8	2.0
Wheat silage	0.0	0.0
Corn silage	9.6	23.1
Oat hay	62.2	8.9
Clover hay	0.9	2.2
Wheat straw	0.0	0.0
Wheat barn	0.0	0.0
DDG ^1^	4.0	9.7
Whey^2^	1.3	3.2
CSFA^3^	0.3	0.8
Urea	0.2	0.4
Salt	0.3	0.7
Oil	0.04	0.1
Sodium bicarbonate	0.4	0.9
Limestone	0.3	0.7
Vitamins and minerals^4^	0.04	0.1
Chemical composition		
NE_L_ (Mcal/kg DM)^5^	1.47	1.78
Crude protein	12.1	16.5
NDF^6^	47.1	29.1
Forage NDF	41.9	16.5
Ether extract	2.9	4.5
Ca	0.009	0.009
P	0.003	0.004

^1^DDG = dried distiller's grains

^2^By-product of the dairy cheese industry

^3^CSFA = calcium salts of palm oil fatty acids

^4^Containing (per kg DM) 20,000,000 IU vitamin A; 2,000,000 IU vitamin D; 15,000 IU vitamin E; 6,000 mg Mn; 6,000 mg Zn; 2,000 mg Fe; 1,500 mg Cu; 120 mg I; 50 mg Se; 20 mg Co.

^5^NE_L_ = net energy for lactation (Mcal/ kg dry matter; NRC 1989 values)

^6^NDF = non-degradable fiber.

Feed samples were analyzed for their content of N (AOAC[31], method 984.13; Kjeltec 8400 Analyzer; Foss Analytical AB, Höganäs, Sweden), NDF and ADF (equipment from Ankom Technology, Fairport, NY), using α-amylase and sodium sulfite for NDF[32], Ca (AOAC[31], method 935.13), P (AOAC[31], method 964.06), and ether extracts (AOAC[31], method 996.06). Samples were dried at 550°C for 3 h for ash determination.

Prepartum, cows were weighed at the day of AT biopsy (14 d prepartum). After calving, milk production and BW were recorded electronically three times daily at the milking parlor (SAE, Kibbutz Afikim, Israel). In addition, milk samples were collected once a month from 3 consecutive milking sessions, and were analyzed for milk fat, protein, lactose, and urea by infrared analysis (standard IDF 141C:2000) at the laboratories of the Israeli Cattle Breeders’ Association (Caesarea, Israel). The body condition score (BCS; scale of 1–5) was determined by a single technician on the day of the AT biopsy: at 14 d prepartum, and at 4 and 30 d PP. According to routine management in Israel, all cows were examined by a veterinarian 5 to 10 d after calving, treated according to the farm's routine management, and clinical events and treatments were recorded.

### Defining the subgroups of cows according to their degree of BW loss PP

To examine the different metabolic responses between PP cows, we divided the cows retrospectively at the end of the study into those with low body weight loss (LWL) and high weight loss (HWL), as described previously[5,7]. Briefly, cows losing more than the mean BW loss of all cows were regarded as HWL and those losing less were defined as LWL. In addition, since we have previously shown that this trait is repetitive within cows across lactations[7], for each of the cows we also collected data on their BW loss in previous lactations, and analyzed the degree of BW loss across lactations.

### Analysis of metabolites and hormones in blood plasma

Blood samples for non-esterified fatty acids (NEFA), insulin, glucagon, glucose, the oxidative stress marker malondialdehyde (MDA), and tumor necrosis factor α (TNFα) were collected twice weekly from 14 d before expected calving until 30 d PP from the jugular vein into vacuum tubes containing lithium heparin (Becton Dickinson Systems, Cowley, England). The blood samples were collected after the morning milking at 0800 h, and plasma was separated after centrifugation at 4000 × *g* for 15 min and then stored at -32°C pending analyses. Plasma NEFA, glucose, insulin, and glucagon were determined as previously described[29] by a single assay; the intra-assay CV was 6.9% for NEFA, 2.6% for glucose, 7.4% for insulin and 7.2% for glucagon. Plasma MDA concentrations were measured according to the fluorimetric thiobarbituric acid reactive substances assay[33]; the intra- and inter-assay CV was 9.6% and 2.7%, respectively. Plasma TNFα concentrations were determined with a Bovine TNF-α duoset ELISA kit (R&D Systems, Inc., Minneapolis, MN); the intra- and inter-assay CV was 9.5 and 5.6%, respectively.

### Adipose tissue biopsies

Subcutaneous AT biopsies were taken from each cow at three time points: late pregnancy (261 ± 5 d of pregnancy, on average, 14 d prepartum), at day 4 PP, and at day 30 PP. The AT samples were taken from the subcutaneous fat pad around the pin bones. Biopsies were taken at each time point from contralateral sides of the tail head region as previously described[5], immediately frozen in liquid nitrogen, and stored at -80°C.

### Endocannabinoid measurements

The levels of eCBs were determined in AT samples obtained at 14 d prepartum (n = 10) and at 4 d PP (n = 10) from a randomly selected subgroup of 5 HWL and 5 LWL cows. AEA, 2-AG, oleoylethanolamide (OEA), palmitoylethanolamide (PEA), and arachidonic acid (AA) in AT were extracted, purified, and quantified by the stable isotope dilution LC-MS/MS method as described previously[34]. The values are expressed as fmol/mg tissue (wet weight), pmol/mg tissue (wet weight), or nmol/mg tissue (wet weight).

### Protein extraction from AT and proteomic analysis

Proteins were extracted from additional AT samples obtained from the same randomly selected subgroup of 5 HWL and 5 LWL cows used for the eCB analysis at 14 d prepartum (n = 10), and at 4 d PP (n = 10) as previously described[29]. Additionally, we measured the proteome of AT in dairy cows 30 d PP (n = 10), since data at this time in lactation have not yet been explored. The protein concentration in each sample was determined using the bicinchoninic acid (BCA) assay. Samples were subjected to in-solution tryptic digestion using a modified Filter Aided Sample Preparation (FASP) protocol and then were loaded onto a nano-ultra performance liquid chromatography apparatus (UPLC), and finally subjected to mass spectrometry as previously described[29]. Protein was quantified by intensity-based label-free proteomics, and raw data were processed as previously described[29,35].

### Bioinformatics analysis

Proteins that were differentially abundant at *P* < 0.05 and fold change (FC) ± 1.5 were analyzed by Qiagen’s Ingenuity^®^ Pathway Analysis (IPA^®^, Qiagen Redwood City, www.qiagen.com/ingenuity) to determine the most significantly relevant pathways and biological functions. Data from IPA were curated for relevance to ruminants by excluding irrelevant pathways.

### Western blot analysis

To validate the results of the proteomic data, protein abundance was determined in AT samples obtained at 14 d prepartum and at 4 d PP. For western blotting, the protein concentration of the sample homogenate was measured according to the Bradford assay (Bio-Rad protein quantification kit). Then, 20 μg of sample in Laemmli loading buffer was resolved by SDS-PAGE under reducing conditions, and transferred onto a nitrocellulose membrane with the following antibodies: perilipin (1:2000, #9349, Cell Signaling Technology, Danvers, MA), MGLL (1:1000, ab24701, Abcam Biotech, Cambridge, UK), and β-actin (1:1000, ab46805, Abcam Biotech). In addition, we examined the abundance of CB1 (1:200, ab23703, Abcam Biotech) and FAAH (1 μg/mL, ARP33121_P050, Aviva Systems Biology, San Diego, CA) in AT samples. Goat anti-rabbit HRP conjugated secondary antibody (Jackson Immunoresearch 111-035-003, PA, USA) at a concentration of 1:10,000 was used for an enhanced chemiluminescence reaction for protein detection. Data were processed and analyzed by densitometry using ImageJ software (NIH, Bethesda, MD). To ensure that quantitative data were obtained, chemiluminescence signals were measured after at least five consecutive exposure times to determine the linear range of the signal intensity of each antibody. Specific band signals were normalized to β-actin.

### Quantitative real-time PCR of AT samples

Gene expression was determined in all AT samples obtained at 14 d prepartum and at 4 d PP (excluding 7 biopsies in which we did not have sufficient biological replicates for mRNA analyses). For RNA extraction, ∼40 mg of AT samples were homogenized with one metal bead in 1 mL of lysis solution using the RNeasy lipid tissue mini kit (Qiagen, Hilden, Germany). RNA quality was assessed and its 260/280 ratio was above 1.85. First-strand cDNA was generated using a cDNA reverse transcription kit (Applied Biosystems, Foster City, CA). Quantitative detection of specific mRNA transcripts was carried out by real-time PCR using a StepOnePlus instrument (Applied Biosystems) using the SYBR green PCR mix (Invitrogen, Carlsbad, CA). We examined the expressions of eCB-related genes: *CNR1*, *CNR2*, *MGLL*, fatty acid amide hydrolase (*FAAH)*, N-acyl phosphatidylethanolamine-specific phospholipase D (*NAPEPLD*); genes related to lipid metabolism: hormone-sensitive lipase (*HSL*), leptin (*LEP*), and fatty acid binding protein 4 (*FABP4*); and inflammatory genes: *TNFα*, interleukin-1β (*IL1β*), and haptoglobin (*HP*). The list of primers is presented in **Table 2**. Primers were validated before use, and data were normalized for the content of the reference gene, bovine ribosomal protein S2 *(BRPS2*) mRNA in AT samples. This gene was chosen as a reference gene after examining several candidate genes in AT (*UXT*, *BRPS2*, *EIF4E*, *GAPDH*, and *β-actin*), and it was concluded that the *BRPS2* expression levels were the most stable ones among the AT samples. The values used for statistical analysis were the delta-delta CT (relative quantity, *RQ*) of each gene; the values were normalized to the quantity of one LWL cow and then divided by the average RQ of each gene in the LWL group (hence, the average RQ in LWL is 1 for each gene).

**Table 2:** List of primers used for mRNA expression in AT.

Gene	GenBank accession no.	Sequence 5`>3`
*CNR1*	NM_001242341.1	F:AAGCCCGCATGGACATTAGGTTAG
		R: AGCAGAGGGCCCCAGCAGAT
*CNR2*^*1*^	NM_001192303.1	F: TCTTCGCCGGCATCATCTAC
		R: CATCCGGGCTATTCCAGACA
*MGLL*^*1*^	NM_001206681.1	F: GCAACCAGCTGCTCAACAC
		R: AGCGTCTTGTCCTGGCTCTT
*FAAH*^*1*^	NM_001099102.2	F: TTCCTGCCAAGCAACATACCT
		R: CACGAAATCACCTTTGAAGTTCTG
*NAPEPLD*^*1*^	NM_001015680.1	F: AGAGATCACAGCAGCGTTCCAT
		R: ACTCCAGCTTCTTCAGGGTCATC
*HSL*	NM_001080220.1	F: GAGTTTGAGCGGATCATTCA
		R: TGAGGCCATGTTTGCTAGAG
*LEP*	NM_173928.2	F: GGCTTTGGCCCTATCTGTCTTA
		R: GAGACGGACTGCGTGTGTGA
*TNFα*	NM_173966.3	F: CCATCAACAGCCCTCTGGTT
		R: GGGCTACCGGCTTGTTACTT
*FABP4*	NM_174314.2	F: GTCATGAATGGTGTCACTGCC
		R: GGAGTTCGATGCAAACGTCA
*BRPS2*	NM_001033613.2	F: GGAGCATCCCTGAAGGATGA
		R: TCCCCGATAGCAACAAACG
*HP*	NM_001040470.2	F: GGTTCGGAAAACCATCGCTA
		R: CACTCGTGTCCCCTCCCTC
*IL1β*	NM_174093.1	F: TCCACCTCCTCTCACAGGAAA
		R: TACCCAAGGCCACAGGAATCT

CNR1 = CB1 receptor, CNR2 = CB2 receptor, MGLL = monoglyceride lipase, FAAH = fatty acid amide hydrolase, NAPEPLD = N-acyl phosphatidylethanolamine-specific phospholipase D, HSL *=* hormone-sensitive lipase, LEP = leptin, FABP4 = fatty acid binding protein 4, TNFα = tumor necrotizing factor α, IL1β = interleukin-1β, HP = haptoglobin, BRPS2 = bovine ribosomal protein S2.

^1^the sequence for this gene was based on Khan et al.[28].

### Statistical analysis

Plasma concentrations of glucose, NEFA, insulin, glucagon, the insulin/glucagon ratio, MDA, and TNFα as well as milk and FCM yields and cows’ body weights, protein abundances from western blots as well as mRNA expressions were analyzed as repeated measurements with the MIXED procedure, version 9.2 (SAS, 2002). BCS measurements at 14 d prepartum, 4 and 30 d PP were analyzed using the SAS GLM procedure (version 9.2, 2002).

The values of eCB measurements are expressed as the mean ± SEM. Unpaired two-tailed Student’s t-test was used to determine differences between groups. In addition, in order to examine the effects of time and the interaction time*subgroup, we analyzed the eCB data as repeated measurements with the MIXED procedure, version 9.2 (SAS, 2002).

Proteomics data, after logarithmic transformation, were analyzed by unpaired two-tailed Student’s t-test to examine the effects of subgroup (HWL vs. LWL) at each time point (14 d prepartum, 4 and 30 d PP). Differentially abundant proteins (DAP) for each effect were determined by *P* < 0.05 and an absolute fold change (FC) > 1.5. In addition, we analyzed the proteomic data by 2-way ANOVA (Statmodel of Python, version 3.6.4) to determine the effects of time and the interaction between time and subgroups (HWL vs. LWL). The data analysis was based on Feise's[36] strategy by calculating the ratios of the observed quantitative difference as well as identifying outlying measurements that would skew the analysis, specifically for every significant protein. Next, we corroborated the findings of several proteins from the proteomic analysis by validation using independent analytical methods (RT-PCR and WB). When FC was greater than +1,000 or less than -1,000, the abundance of the specific protein was considered as present in one group and not detected in the other.

The effect of disease was examined and was not found to be significant for any of the variables; therefore, it was excluded from the model. Significance was set at *P* < 0.05 and tendencies were set at *P* < 0.10.

## Results

### Characterizing cows as HWL and LWL PP

**Fig 1:**
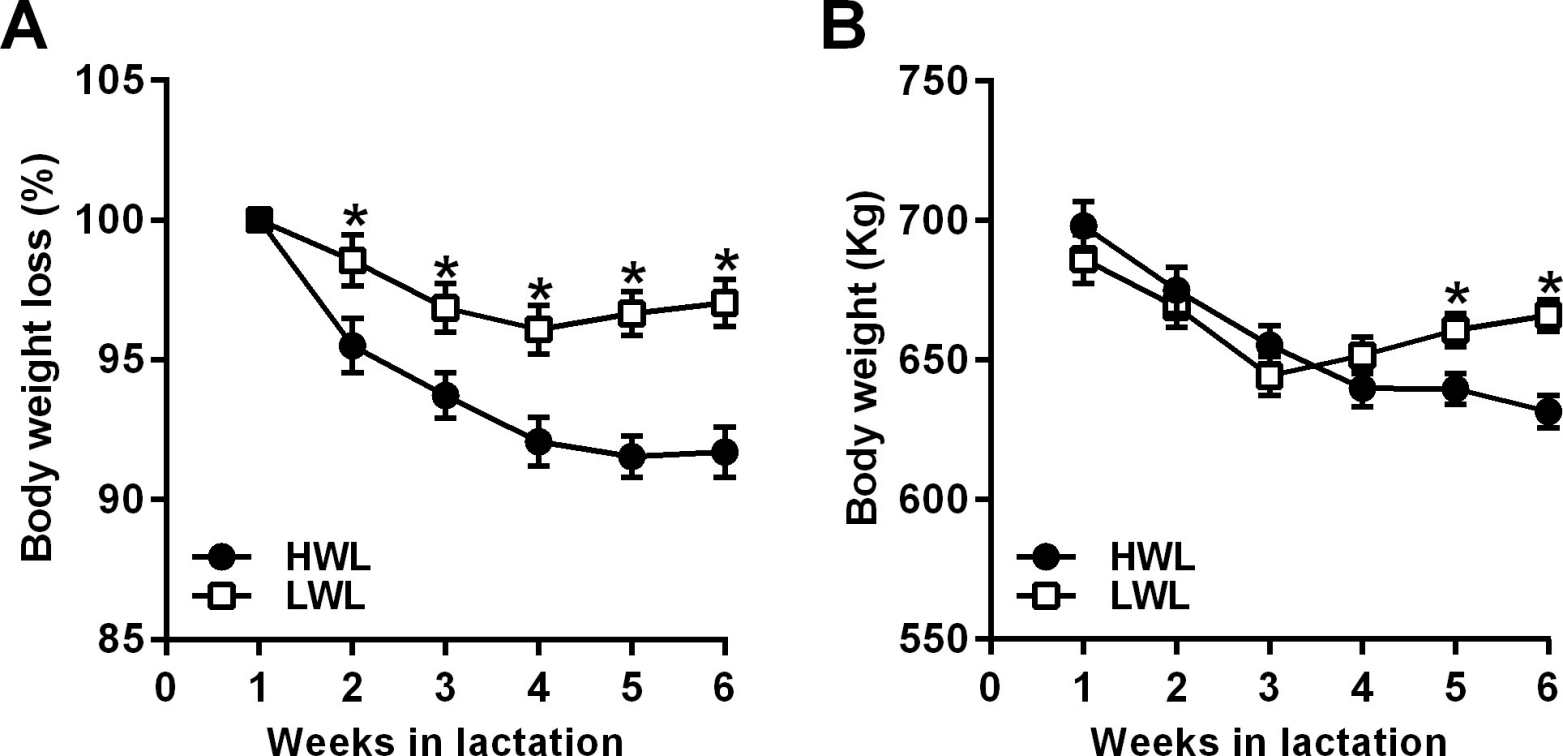
**Percentage of BW compared to week 1 postpartum (A) and the average BW (B) in HWL and LWL dairy cows.** Cows were categorized either as high-weight loss (HWL, n = 9) or low-weight loss (LWL, n = 9), based on the percentage of BW loss between week 1 and 5 postpartum. Weekly averages of the percentage of BW compared to week 1 postpartum (**A**), and weekly averages of BW (**B**) are presented ± SEM. *P<0.05 in HWL compared to LWL; +*P*<0.1 in HWL compared to LWL cows.

In the present study, we retrospectively characterized the cows as HWL or LWL based on the percentage of BW loss between week 1 and week 5 PP, as previously described[5,7]. Accordingly, nine cows were defined as HWL (on average, 8.5 ± 1.7% BW loss, range 6.8–11.5%), and 9 cows were LWL (on average, 2.9 ± 2.5% BW loss, range -0.6–6%). The average lactation number was similar between groups: 3.6 lactations for HWL and 3.7 lactations for LWL cows (range: 2–6 lactations). As shown in Fig 1A, HWL cows lost significantly more BW (as a percentage of BW at week 1 PP) than did LWL cows between weeks 2–6 PP (Fig 1A). The weekly average BW of each subgroup during weeks 1–6 PP is shown in Fig 1B; the average BW of HWL cows at week 1 PP tended to be higher than LWL (703.3 vs. 680.6 kg, for HWL and LWL, respectively, SEM = 8.7, *P* < 0.07), as well as in week 2 PP (689.2 vs. 669.1 kg, for HWL and LWL, respectively, SEM = 7.8, *P* < 0.07). However, the average BW was significantly lower in HWL than in LWL in weeks 5 and 6 PP (*P* < 0.01; Fig 1B). Importantly, the average BW at 14 days before calving was not different between groups (737.9 and 719.1 kg, for HWL and LWL, respectively, SEM = 23.2, *P* < 0.6). To further support our decision to analyze the present study based on the degree of BW loss during the first month PP, we collected the BW data from the previous lactations of the 18 cows participating in this study, and analyzed the percentage of BW loss across lactations. Interestingly, it was found that across lactations 2–6, the percentage of BW loss tended to be higher in HWL than in LWL cows (8.6 ± 0.9% in HWL vs. 5.7 ± 1.4% in LWL, *P* < 0.1), indicating that this trait is repetitive within animals and reflects the difference in metabolic response PP.

The cows' body condition score (BCS), an indicator of their degree of fatness, was near tendency to be higher in HWL compared with LWL cows at 14 d prepartum (3.9 vs. 3.5 units, SEM = 0.2, *P* < 0.13). No differences in BCS between groups were observed at 4 d PP (3.4 and 3.1 units in HWL and LWL cows, respectively, SEM = 0.2, *P* < 0.3). However, the loss of BCS units from 14 d prepartum to 30 d PP tended to be higher in HWL compared with LWL cows (1.4 vs. 0.9 units in HWL and LWL cows, respectively, SEM = 0.18, *P* < 0.07).

Milk yields during the first month PP did not differ between HWL and LWL cows (36.0 and 37.7 kg/d, respectively, SEM = 2.3, *P* < 0.6), nor did the 4% fat-corrected milk (FCM) production (34.0 and 37.4 kg/d, SEM = 3.0, *P* < 0.4). In addition, milk yields during the first 100 d of lactation did not differ between HWL and LWL cows (42.1 and 41.6 kg/d, SEM = 2.9, *P* < 0.9) nor did the 4% FCM production (38.0 and 39.2 kg/d, SEM = 3.1, *P* < 0.8). Information about the cows' feed intake is lacking due to the group housing of the animals in this experiment. The average THI during the experiment was 72.4 ± 3.4 (range 65.4–78.1).

None of the cows showed any clinical signs of disease on the day of the AT biopsy. All cows were examined by a veterinarian at 5–10 d PP according to the farm’s routine; one HWL and one LWL cow had light ketosis and they were treated once with a propylene glycol drench and fully recovered. Two other cows (1 HWL and 1 LWL) were detected with metritis; they were successfully treated once and recovered.

### Circulating levels of metabolites and hormones in HWL and LWL cows

**Fig 2:**
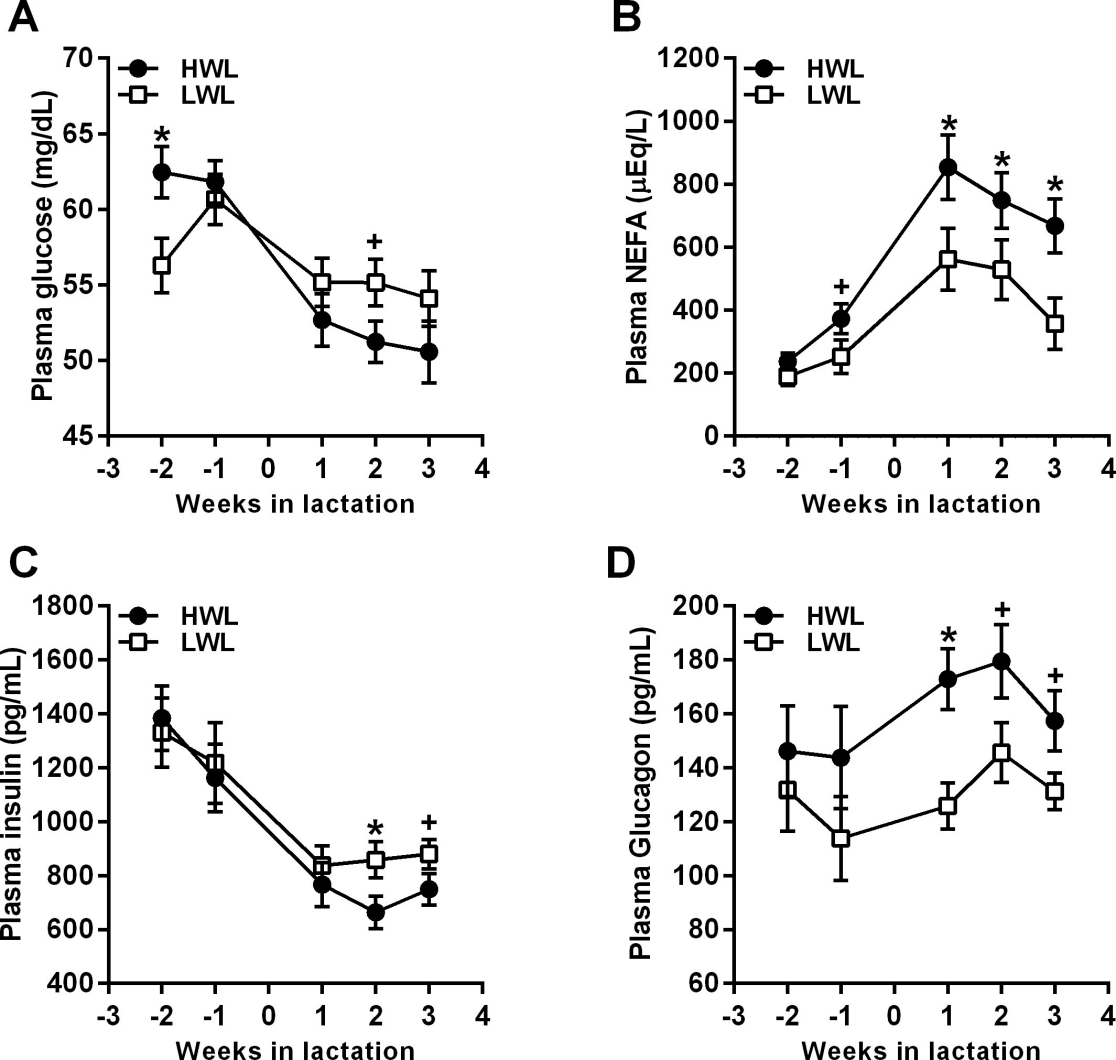
**Weekly average concentrations of plasma glucose (A), NEFA (B), insulin (C), and glucagon (D) in HWL and LWL cows.** Cows were categorized either as high-weight loss (HWL, n = 9) or low-weight loss (LWL, n = 9), based on the percentage of BW loss between week 1 and 5 postpartum. Data represent the mean ± SEM. *P<0.05 in HWL compared to LWL; +*P*<0.1 in HWL compared to LWL cows.

Prepartum, glucose levels were higher in HWL than in LWL cows (61.8 vs. 58.1 mg/dL, SEM = 1.2, *P* < 0.05; Fig 2A). During the last week of pregnancy, the average plasma NEFA concentrations tended to be higher in HWL compared with LWL cows (369.3 vs. 247.4 μmol/L, respectively, SEM = 50.5, *P* = 0.1; Fig 2B). No differences in plasma insulin (*P* < 0.7; Fig 2C), glucagon (*P* < 0.3; Fig 2D), and in the insulin/glucagon ratio (*P* < 0.3) were observed prepartum between HWL and LWL cows.

Postpartum, plasma glucose concentrations tended to be lower in HWL than in LWL cows (51.3 vs. 54.2 mg/dL in HWL and LWL, respectively, SEM = 1.3, *P* < 0.1; Fig 2A), and plasma levels of NEFA were significantly higher in HWL than in LWL cows PP (636.1 vs. 454.6 μmol/L, respectively, SEM = 47.2, *P* < 0.02; Fig 2B). Plasma insulin concentrations tended to be lower in HWL than in LWL cows PP (733.6 and 866.0 pg/mL in HWL and LWL, respectively, SEM = 53.6, *P* < 0.1; Fig 2C). Glucagon levels were higher in HWL compared with LWL cows PP (171.0 vs. 134.1 pg/mL, respectively, SEM = 10.3, *P* < 0.05; Fig 2D), and the insulin/glucagon ratio was significantly lower in HWL than in LWL PP cows (4.1 vs. 6.0 in HWL and LWL, respectively, SEM = 0.5, *P* < 0.04).

Since increased lipolysis in AT elevates oxidative stress[37], we examined the circulating levels of MDA, an indicator of oxidative stress. No differences in MDA levels were observed between HWL and LWL cows prepartum (330.4 and 242.1 nM, respectively, SEM = 54.2, *P* < 0.3) or PP (430.4 and 325.0 nM, respectively, SEM = 53.0, *P* < 0.2). However, the MDA levels tended to be higher in HWL compared with LWL cows during the first week PP (474.2 vs. 324.2 nM, respectively, SEM = 65, *P* < 0.1). Throughout the transition period, plasma TNFα levels were similar in HWL vs. LWL cows (663 vs. 353 pg/mL, respectively, SEM = 230, *P* < 0.4).

#### eCB levels in AT

**Fig 3:**
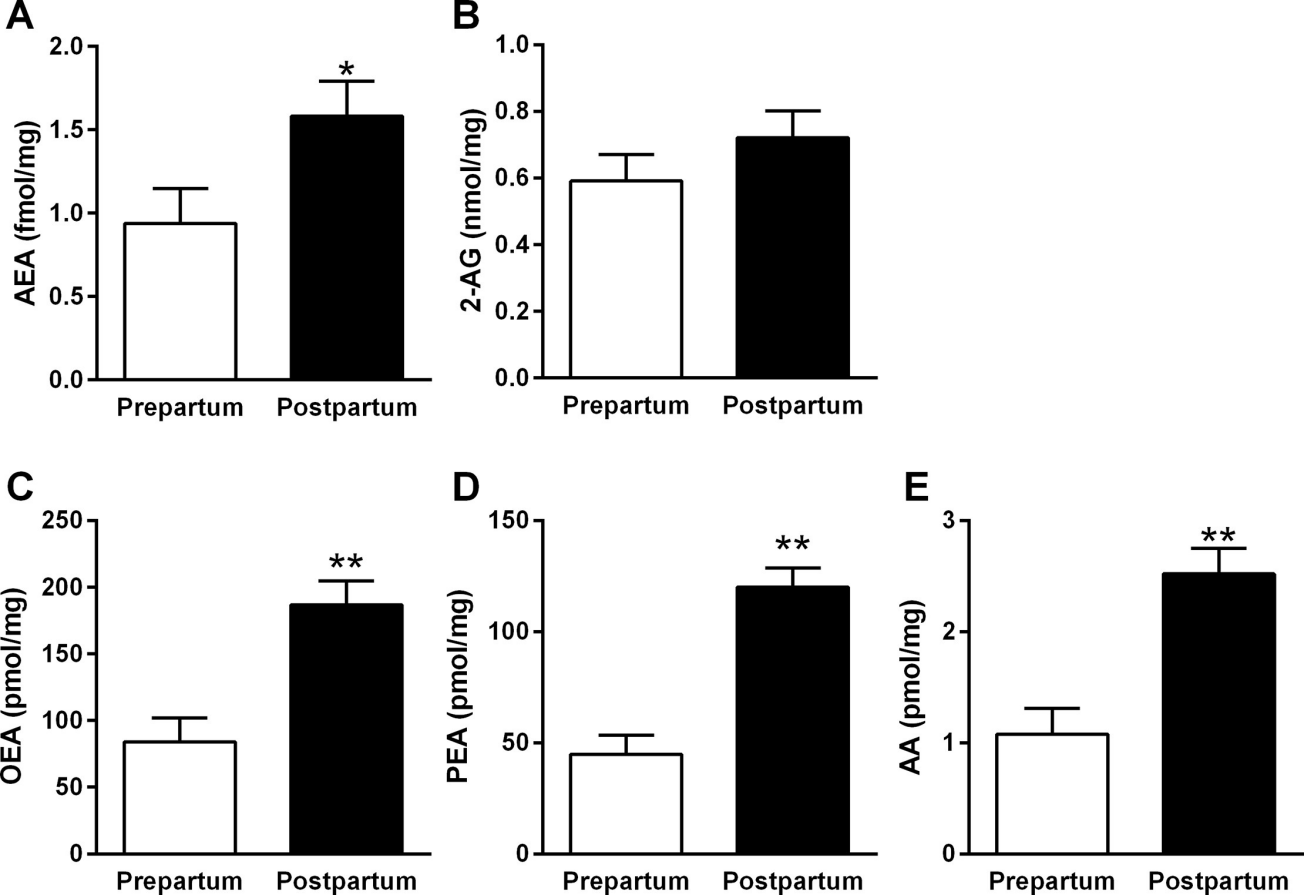
Levels of AT eCBs that differed pre- and PP in the AT of dairy cows. AT was sampled at 14 d prepartum (n = 10) and at 4 d PP (n = 10) for levels of AEA (**A**), 2-AG (**B**), OEA (**C**), PEA (**D**), and AA (**E**). Data represent the mean ± SEM.

A comparison of eCB levels in pre- vs. PP AT revealed elevated levels of eCB PP vs. prepartum: AEA (1.58 vs. 0.93 fmol/mg, SEM = 0.21, *P* < 0.04; Fig 3A), OEA (186.7 vs. 84.0 pmol/mg, SEM = 18.0, *P* < 0.0003; Fig 3C), PEA (120.2 vs. 44.8 pmol/mg, SEM = 8.6, *P* < 0.0001; Fig 3D), and AA (2.5 vs. 1.1 nmol/mg, SEM = 0.2, *P* < 0.0001; Fig 3E), respectively. The levels of 2-AG in AT were similar PP compared to those levels prepartum (0.72 vs. 0.59 nmol/mg, respectively, SEM = 0.08, *P* < 0.3; Fig 3B).

**Fig 4:**
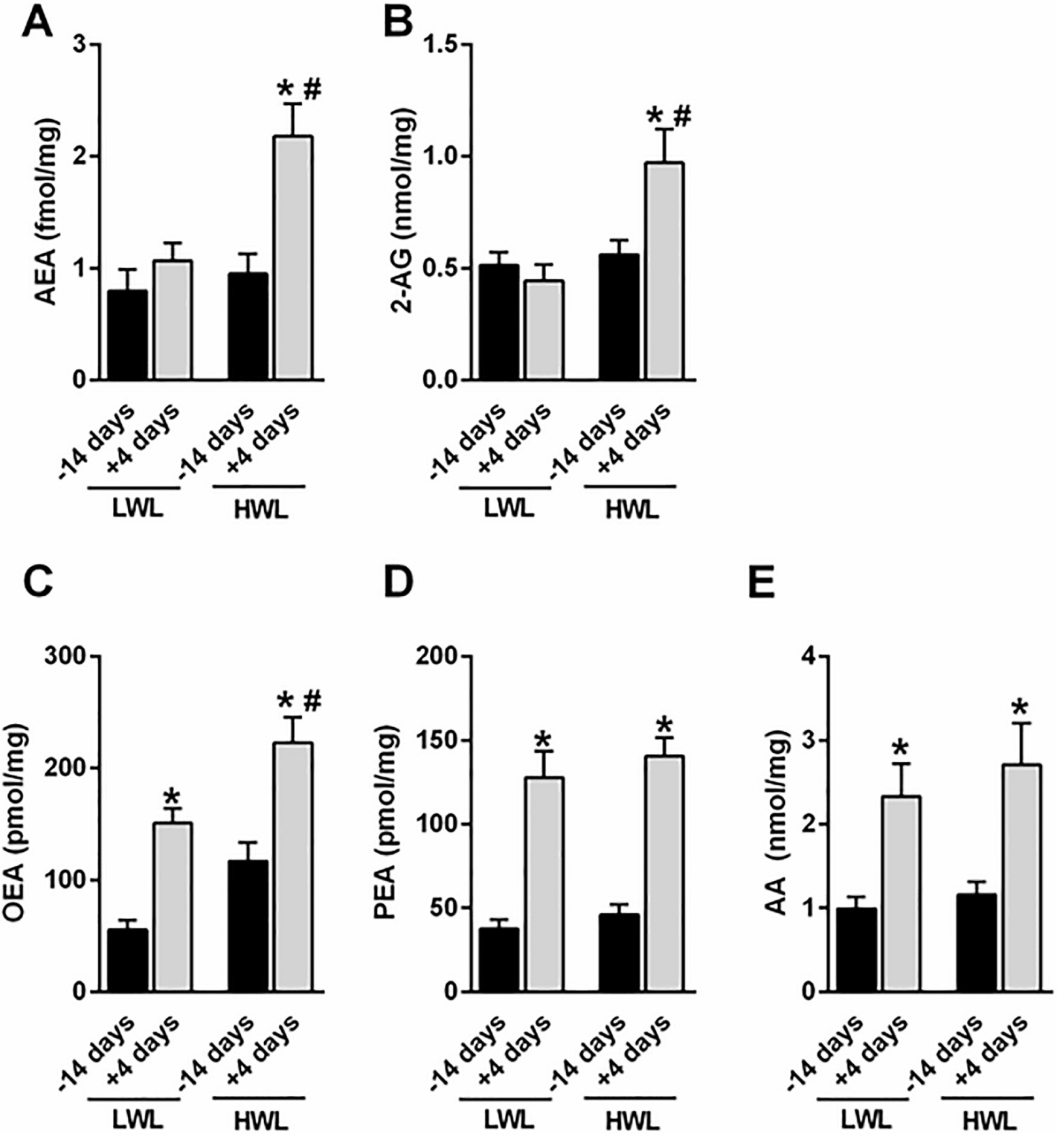
Levels of eCBs in the AT of HWL and LWL dairy cows. Cows were categorized as either high-weight loss (HWL, n = 9) or low-weight loss (LWL, n = 9), based on the percentage of BW loss between week 1 and 5 postpartum. Arachidonic acid (AA), 2-arachidonoylglycerol (2-AG), anandamide (AEA), oleoylethanolamide (OEA), and palmitoylethanolamide (PEA). Data represent the mean ± SEM. *P<0.05 vs. -14 days of the same group. #*P*<0.05 vs. +4 days of the LWL cows.

When we compared eCB levels in HWL vs. LWL AT, we found twofold elevated levels of AEA (Fig 4A) and 2-AG (Fig 4B) in AT of HWL cows at day 4 PP compared with 14 d prepartum (AEA: 0.94 vs. 2.18 fmol/mg, in pre- and PP, respectively, SEM = 0.23, *P* < 0.05; 2-AG: 0.56 vs. 0.97 nmol/mg, in pre- and PP, respectively, SEM = 0.10, *P* < 0.05), but those differences between pre- and PP were not found in LWL cows. On the other hand, the levels of OEA, PEA, and AA were significantly higher in PP compared with prepartum AT, both in LWL and HWL cows (Fig 4C–4E). In addition, a significant difference was found in OEA levels at day 4 PP between LWL and HWL cows (Fig 4C; 151.0 vs. 223.0 pmol/mg, SEM = 17.9, *P* < 0.05). Except for 2-AG (*P* < 0.01), the interaction of time and subgroup (HWL vs. LWL) was not significant.

#### Gene expressions in AT

The expressions of genes related to the eCB system, lipid metabolism, and inflammation were examined in pre- and PP AT. Prepartum, the expression of *CNR1*, encoding the CB1 receptor, tended to be higher in HWL compared with LWL AT (1.0 vs. 0.1 RQ in HWL and LWL, respectively, *P* < 0.07), and the expression of *CNR2*, encoding the CB2 receptor, did not differ between the subgroups (1.0 and 0.9 RQ in HWL and LWL, respectively, *P* < 0.9). The mRNA expressions of eCB-related genes: *MGLL*, *FAAH*, and *NAPEPLD*; of the lipid metabolism genes: *HSL*, *LEP*, and *FABP4*, and of inflammatory genes: *TNFα*, *IL1β*, and *HP* did not differ between subgroups in prepartum AT (*P* < 0.8).

Postpartum, when differences in eCBs were evident between HWL and LWL AT, the mRNA expressions of *CNR1* (*P* < 0.1) and of *CNR2* (*P* < 0.1) in AT tended to be higher in HWL compared with LWL cows (Table 3). Across times (14 d prepartum and 4 d PP), the expression of *CNR1* tended to be higher in HWL than in LWL cows (1.0 vs. 9.9 RQ for LWL and HWL, respectively, SEM = 4.1, *P* < 0.1). The mRNA expression levels of *MGLL*, *FAAH*, *NAPEPLD*, *HSL*, *LEP*, *FABP4*, *TNFα*, and *IL1β* did not differ between the subgroups, but the expression of HP tended to be higher in HWL vs. LWL AT PP (*P* < 0.1; Table 3). The effects of time and the interaction of time and subgroup (HWL vs. LWL) were not significant for any of the examined genes.

**Table 3:** Relative mRNA abundance (fold) in AT from HWL and LWL cows at 4 d PP.

Gene, RQ^1^	HWL	LWL	SEM	*P* <	Efficiency, %^2^
CNR1	13.0*	1.0	5.41	0.1	100
CNR2	22.6*	1.0	8.50	0.1	100
FAAH	1.0	1.0	0.17	1.0	100
MGLL	2.9	1.0	1.16	0.3	92
NAPEPLD	1.6	1.0	0.59	0.4	100
HSL	1.1	1.0	0.30	0.8	90
IL1β	11.9	1.0	5.56	0.2	90
LEP	0.9	1.0	0.51	0.9	79
FABP4	0.7	1.0	0.34	0.6	91
HP	50.0*	1.0	22.1	0.1	100
TNFα	4.6	1.0	2.4	0.3	100

^1^RQ = delta-delta CT values were normalized to the average value of the LWL subgroup for each gene.

CNR1 = CB1 receptor, CNR2 = CB2 receptor, MGLL = monoglyceride lipase, FAAH = fatty acid amide hydrolase, NAPEPLD = N-acyl phosphatidylethanolamine-specific phospholipase D, HSL *=* hormone-sensitive lipase, LEP = leptin, FABP4 = fatty acid binding protein 4, TNFα = tumor necrotizing factor α, IL1β = interleukin-1β, HP = haptoglobin.

*P < 0.1 between HWL and LWL AT PP.

^2^The efficiencies were calculated according to the standard curve of each primer with different cDNA concentrations; the efficiency of the reference gene BRPS2 was 100%.

### Proteomic analysis of ATs

To explore the shift in the AT metabolic state, we conducted a longitudinal proteomic analysis on AT samples from HWL and LWL cows (n = 5 in each) at 3 time points for each cow: 14 d prepartum, 4 d, and 30 d PP (a total of 30 AT samples were analyzed). A total of 1,913 proteins were identified in AT (S1 Table). Since the objective of the proteomic analysis was to examine differences in the AT metabolic state between HWL and LWL, we focused on the effect of subgrouping at each time point. The abundance of 28 proteins was different [*P* < 0.05 and fold change (FC) ± 1.5] between HWL and LWL AT at 14 d prepartum, whereas 176 proteins (9.2% of total proteins) differed between HWL and LWL AT at 4 d PP, and 35 proteins differed between HWL and LWL AT at 30 d PP. Of note, the dataset of the AT proteome at 14 d prepartum was previously applied to examine seasonal effects[29].

**Fig 5:**
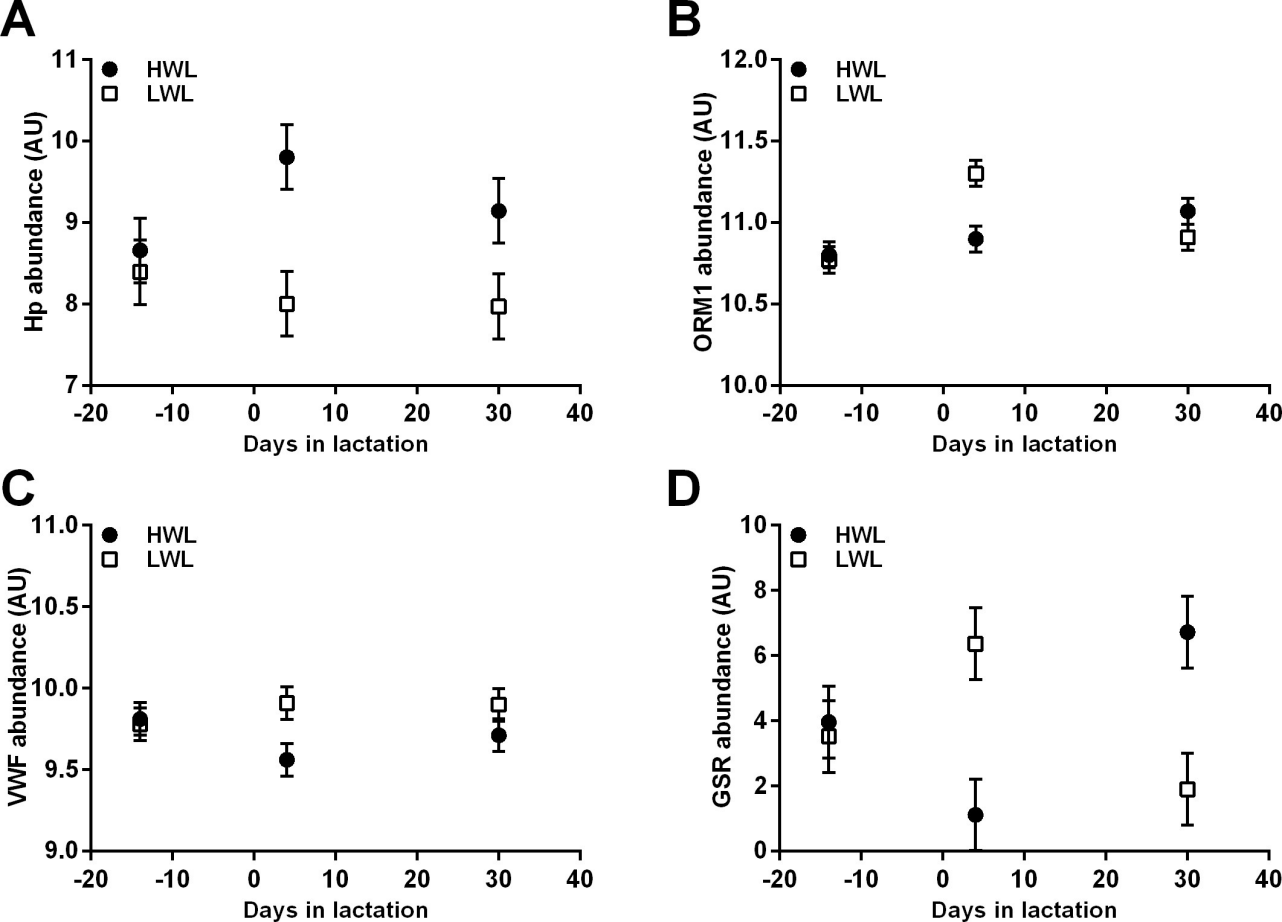
Longitudinal differential abundances of acute phase and oxidative stress-related proteins in AT of HWL and LWL cows according to proteomic analysis. Cows were categorized either as high-weight loss (HWL, n = 9) or low-weight loss (LWL, n = 9) based on the percentage of BW loss between weeks 1 and 5 postpartum. The biopsies of subcutaneous AT from a subgroup of HWL and LWL AT (n = 5 per group) from 14 d prepartum, 4 d, and 30 d postpartum were analyzed by proteomic analysis. HP = haptoglobin; ORM1 = alpha-1-acid glycoprotein; VWF = von Willebrand factor; GSR = glutathione reductase.

Comparing the top 25 canonical pathways by Venn diagram[38] (S1 Fig) revealed that two pathways were enriched at all 3 time points in HWL vs. LWL AT: the complement system and acute phase response signaling. Indeed, the abundances of three acute phase proteins differed in AT of HWL compared with LWL cows at 4 d PP: haptoglobin (HP; FC = 64, *P* < 0.006; Fig 5A), alpha-1-acid glycoprotein (ORM1; FC = -2.35, *P* < 0.007; Fig 5B), and von Willebrand factor (VWF; FC = -2.33, *P* < 0.01; Fig 5C). The abundance of the oxidative stress-related protein glutathione reductase (GSR) was not detected in HWL but was abundant in LWL AT at 4 d (FC < 1000; *P* < 0.037; Fig 5D), whereas at 30 d it showed an opposite trend (FC > 1000; *P* < 0.02; Fig 5D). The effects of time and the interaction of time and subgroup were not significant for HP, VWF, or GSR. However, the effect of time (*P* < 0.03) and the interaction of time and subgroup (*P* < 0.004) were significant for ORM1.

**Fig 6:**
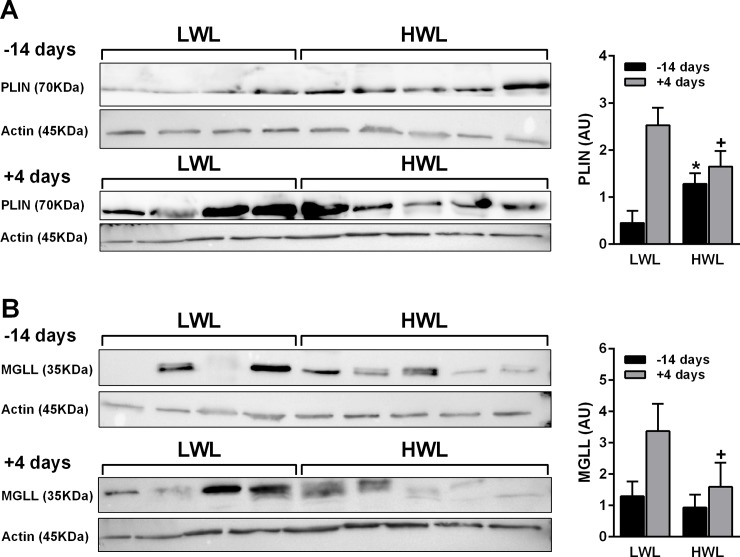
Protein expression of PLIN1 and MGLL in ATs collected at 14 d prepartum and 4 d PP from HWL and LWL cows. Cows were categorized as either high-weight loss (HWL, n = 9) or low-weight loss (LWL, n = 9) based on the percentage of BW loss between weeks 1 and 5 postpartum. The protein abundances of PLIN1 (**A**) and MGLL (**B**) were assessed by Western blotting analysis and corrected by β-actin as an internal standard. Samples for the western blot were prepared (×3.5), divided, and loaded to separate gels that ran simultaneously. The MGLL, PLIN1, and β-actin ran in parallel gels due to the proximity of the bands. Data represent the mean ± SEM. *P<0.05 in HWL vs. LWL AT prepartum. +*P*<0.1 in HWL vs. LWL AT postpartum.

The abundance of the lipid metabolism-related protein perilipin 1 (PLIN1) tended to be lower in HWL than in LWL at 4 d PP (FC = -2.03, *P* < 0.09), which was validated by a western blot analysis (*P* < 0.1; Fig 6A). The abundance of MGLL did not differ between HWL and LWL AT at different time points, but after combining the proteomic data from 14 d prepartum with 4 d PP (across times), the abundance of MGLL was lower in HWL than in LWL AT (*P* < 0.04). This was validated by a western blot analysis that showed a trend for reduced abundance of MGLL in HWL than in LWL AT PP (*P* < 0.17, Fig 6B). For MGLL in WB, across times, the effect of subgroup (HWL vs. LWL) tended to be significant (*P* < 0.08); the effect of time tended to be significant (*P* < 0.09), but there was no interaction of time and subgroup. In the proteomic data, the effects of time and the interaction of time and subgroup were not significant for these proteins.

The proteins CB1 and FAAH were not detected by the proteomic analysis; therefore, we examined their abundance in AT by Western blots. Although both the CB1 receptor and FAAH were clearly abundant in all ATs, no differences were found between HWL and LWL AT either prepartum (*P* < 0.7), or PP (*P* < 0.98; S2 Fig). No differences in the abundance of CB1 (*P* < 0.4) and FAAH (*P* < 0.7) were found when comparing prepartum to PP AT in all cows. The effects of time and the interaction of time and subgroup (HWL vs. LWL) were not significant for these proteins.

## Discussion

To the best of our knowledge, this is the first study to describe the presence of key elements of the eCB system in subcutaneous AT in dairy cows. We found elevated levels of eCB AEA, which is a key regulator of metabolism and energy homeostasis, and of the eCB-like molecules PEA, OEA, and AA, in PP compared with prepartum AT. After analyzing the results by the degree of BW loss PP, increased stimulation of the eCB system was found in HWL cows, reflected by: i) elevated levels of AEA and 2-AG, ii) reduced protein abundance of MGLL, and iii) a tendency for elevated gene expression levels of *CNR1* and *CNR2* in PP AT, compared with LWL cows. This may be in accordance with the differential metabolic state of these cows, as reflected by blood metabolites and hormones. In addition, the longitudinal proteomic analysis of AT revealed increased inflammation through enrichment of the complement and acute phase signaling pathways in PP AT of HWL cows, suggesting that increased eCB stimulation may be related to elevated lipolysis levels and that it is associated with inflammation in AT. This stimulation of the eCB system during the early PP period seems to be part of the metabolic adaptations related to the onset of lactation.

In the present study we found that the levels of AEA and 2-AG were elevated 2-fold PP compared with prepartum in AT of HWL cows, but not in LWL cows. These striking differences were quite surprising, since the levels of OEA, PEA, and AA in AT were higher PP compared with prepartum in all cows. In humans, insulin negatively regulates eCB levels[39]; therefore, the tendency for lower plasma insulin levels in HWL cows PP may be related to the increased production of eCBs. The levels of AA, the main degrading product of both AEA and 2-AG, were elevated PP similarly in HWL and LWL cows. Hence, we would have expected to find no differences in the eCB levels of the LWL and HWL cows. However, we found increased eCB levels (2-AG and AEA) in HWL AT at 4 d PP, and the levels of the endogenous fatty acid amides (or eCB-like molecules), OEA, and PEA were higher PP in all cows. These ligands, structurally very similar to AEA, and which share common biosynthesis and degradation pathways, are not classical eCBs, since they do not bind to the CB1 and CB2 receptors. This implies that they do not contribute to the development of metabolic abnormalities seen in the HWL cows. Nevertheless, since OEA signals via PPARα and mediates anti-lipogenic effects[40], it is tempting to suggest that it may also contribute to the increased BW loss seen in the HWL cows, which exhibited higher OEA levels in AT at 4 d PP. This is in accordance with elevated adipocyte OEA levels and decreased subcutaneous fat content that was observed in obese, type-2 diabetic patients [41]. It should be noted that BW loss PP is a normal physiological process in high-yielding PP dairy cows, and that the BW loss in the HWL cows was within a normal range and not indicative of a pathological state. Although sharing a similar inactivating enzymatic pathway (via FAAH), the adipose AEA, OEA, and PEA levels did not behave similarly PP. Whereas AEA was not elevated in AT of LWL at 4 d PP, OEA and PEA were significantly upregulated at the same time point. It is therefore reasonable to assume that OEA and PEA in LWL cows may contribute to their altered metabolic profile and reduced AT inflammation and lipolysis.

Endogenous AEA plays a major role in the development of diet-induced obesity and fatty liver in rodents[42]; more specifically, it stimulates food intake[43] and energy storage in the form of lipids[44]. In contrast, OEA is anorexigenic and enhances AT lipolysis[45]. In the present study, the elevated levels of AEA, 2-AG, and OEA in PP AT of HWL cows, which exhibit increased lipolysis, is in accordance with a report of elevated 2-AG levels during weight loss[46]. However, this is in contrast with other studies reporting hyperactivity of the eCB system during obesity (reviewed in [14]) and with the role of AEA in lipid storage. In contrast, reduced subcutaneous AT 2-AG and/or AEA levels were also reported in obese rodents[47,48]. Moreover, since it was suggested that alterations in eCB levels in humans with obesity may occur in both a gender- and white-AT-depot-specific as well as in an insulin-dependent manner[14], it is reasonable to assume that this might also be the case with dairy cows, meaning that AT eCB levels reflect differences in metabolic function.

As mentioned above, the only other study that has examined the different components of the eCB system in transition dairy cows found increased hepatic mRNA expression of *CNR2* and *MGLL* in cows fed a moderate-energy diet, compared with those fed a low-energy diet at 7 d PP[28]. Here, we found a tendency for increased AT mRNA expression of *CNR1* in HWL compared with LWL cows at 4 d PP. Overactivation of the eCB/CB1 receptor system has been documented both centrally and peripherally during obesity and IR[26,49,50]. In humans, subcutaneous AT *CNR1* expression levels were 2-fold higher in type 2 diabetic subjects compared with controls[49], suggesting a potential role of the peripheral eCB system in promoting AT dysfunction and inflammation. Conversely, and as mentioned previously, all the biological actions reported for CB1 receptor activation in AT *in vitro* minimize lipolysis[14], which apparently is in contrast with the tendency for increased expression of AT *CNR1* in HWL cows, which exhibited increased lipolysis compared with LWL cows, although we did not examine receptor activation—only gene expression. Moreover, at the protein level, no differences in the abundance of CB1 were detected when comparing HWL and LWL AT both pre- and PP. It can be postulated that the tendency of increase in CB1 expression in HWL AT was a protective mechanism to counter lipolysis. Clearly, further investigation is still required to elucidate the role of the CB1 receptor in AT of PP dairy cows.

A tendency for increased mRNA expression of *CNR2* in AT of HWL compared with LWL cows at 4 d PP was also found. In rodents, *CNR2* expression was found in the epididymal AT of lean animals, and its expression was enhanced in obese mice in parallel to body weight increase. In addition, its induction in AT has been shown to correlate with AT inflammation[51]. Interestingly, it was found that *CNR2* expression was predominantly expressed in the stromal vascular fraction of AT, both in lean and in obese animals, but it was barely detectable in the adipocyte fraction[51]. This is in agreement with the known role of *CNR2* in inflammation, and may suggest that in the AT examined in our study the source of *CNR2* in AT was the stromal vascular fraction and perhaps not the adipocytes.

The abundance of the eCB-degrading enzyme, MGLL, was lower in HWL than in LWL AT across times (prepatrum and PP). In non-ruminants, MGLL plays a key role in degrading and hydrolyzing 2-AG[52]. Indeed, we found that 2-AG levels were higher PP in HWL compared with LWL AT, which could result from the reduced MGLL levels in AT during this time in HWL cows. However, Cable and colleagues[8] concluded that in humans the MGLL activity is not affected by diabetes, dyslipidemia, or other markers of metabolic dysfunction, suggesting that elevated levels of eCBs are not entirely the result of altered degradation in AT. This may suggest that the lower MGLL levels in AT do not directly relate to the levels of eCBs in AT, or alternatively, that the activity of MGLL, and not its abundance, is relevant in this case. Moreover, no differences in the AT expression or the protein abundance of another eCB enzyme, FAAH, were found when examining HWL and LWL AT both pre- and PP. This may imply that MGLL, but not FAAH, is important for the alteration in eCB levels in AT of peripartum cows.

In this study, the metabolic state of HWL cows differed compared to that of LWL cows, as reflected by the changes in circulating metabolites and hormones during the pre- and PP periods. The HWL cows had increased glucose concentrations prepartum and a tendency for reduced glucose levels PP, coupled with a lower insulin-to-glucagon ratio PP compared with LWL cows. Hammon and colleagues[4] observed no differences in plasma glucagon but found a higher glucagon-to-insulin ratio in cows with high compared with low liver fat content, which is in accordance with our current findings, since cows with lower AT lipolysis levels are presumed to have lower liver fat content. This altered metabolic profile of HWL cows during the transition period demonstrates their unique metabolic response, which supports our classification of subgroups of cows.

Lipolysis triggers AT remodeling characterized by enhanced humoral and cell-mediated inflammatory responses and changes in its distribution of cellular populations and extracellular matrix composition[53]. The HWL cows had increased plasma NEFA concentrations compared with LWL PP, which is in accordance with our previous work[6,7] and with the results of Hammom and colleagues[4]; this is expected in cows with higher AT lipolysis. The HWL cows tended to lose more BCS units, ranging from 14 to 30 d PP, than the LWL cows did, which is in agreement with the increased BW loss and increased plasma NEFA concentrations. Since fat mobilization usually increases oxidative stress[37], a tendency for elevated plasma MDA levels was found at week 1 PP in the HWL cows, which is also in agreement with our previous findings[7]. The enrichment in complementary and acute phase protein signaling pathways found by the proteomic analysis of HWL AT suggests increased inflammation in their AT. In support of this, the circulating TNFα levels in HWL cows were higher than in their LWL counterparts throughout the transition period (*P* < 0.05), found in a companion study with a similar design (unpublished data). Recently, increased relative mRNA expressions of the eCB synthesizing enzyme NAPEPLD and of the CNR2 receptor, as well as reduced mRNA expressions of the eCB hydrolyzing enzymes, N-acylethanolamine acid amidase and FAAH, were found in endometrium; this was associated with the inflammatory state of these cows[54]. This suggests that tissue eCBs may be increased during inflammation, which is in agreement with our findings of increased eCBs related to inflammation and lipolysis in AT of PP HWL cows. Regarding AT inflammation, this is a normal physiological state in PP dairy cows, and it is not defined as a pathological process *per se*[37,53].

Since the proteomic analysis was not the main objective of this study, we limited our discussion to the major findings that relate to the metabolic profile of AT in HWL and LWL cows. Interestingly, the two pathways that were enriched at all time points (14 d prepartum, 4 and 30 d PP) in HWL vs. LWL AT were complementary and acute phase signaling. Regarding the acute phase signaling pathway, we found that the abundance of HP was higher in HWL at 4 d PP, whereas that of ORM1 and VWF were lower than in LWL AT. Since ORM1 has an anti-inflammatory function[55], it was suggested that its expression in cows' AT may exert local activity by protecting the tissue from increased oxidative stress[56]. On the other hand, we have previously demonstrated that ORM1 was reduced in AT of prepartum cows calving in summer vs. winter[29]. Hence, its abundance is reduced during elevated oxidative stress[29]; this is in agreement with the present finding since HWL AT was subjected to increased lipolysis, which is associated with higher oxidative stress, which was possibly supported by a tendency for higher plasma MDA during week 1 PP. Since eCBs via the CB1 receptor increase oxidative stress in many tissues, perhaps the reduced ORM1 is associated with increased eCB tone in HWL cows. The reduced abundance of VWF in HWL AT may be for the same reason, whereas the increased HP abundance, which was in agreement with the tendency for increased mRNA expression, can be explained by the elevated oxidative stress[57]. The enrichment of the acute phase signaling pathway indicates an increased inflammatory tone of AT in HWL cows PP.

Taken together, elevated eCB levels in AT were found PP compared with prepartum in dairy cows. Since the eCB system regulates energy homeostasis, these findings may suggest that its role in the metabolic adaptations of the cows is associated with the onset of lactation. Moreover, HWL cows exhibited increased activation of the eCB system in AT coupled with higher lipolysis and signs of increased AT inflammation. Since the HWL cows had similar milk production, we hypothesize that one of the possible physiological outcomes of increased eCB system activation may be related to a hampered reproductive function in these animals[7]; indeed, a link between the eCB system and reproduction in ruminants has been established[58,59]. Clearly, further research is required to investigate the relationship between the eCB system, metabolic function, and reproduction in dairy cows. Future research should attempt to shift the activation of the eCB system, most practically by nutritional manipulations, which will affect either the energy balance or the endogenous levels of eCB in transition cows.

## Supporting information

S1 Table Raw data of proteomic analysis. AT samples from high-weight loss (HWL, n = 5) or low-weight loss (LWL, n = 5) cows, based on the percentage of BW loss between week 1 and 5 postpartum, were analyzed by proteomic analysis at 14 d prepartum, 4 and 30 d PP.
(XLSX)

S1 Fig Venn diagram of the top 25 canonical pathways in AT at 14 d prepartum, 4 d and 30 d postpartum according to the differentially abundant proteins in HWL and LWL dairy cows. AT samples from high-weight loss (HWL, n = 5) or low-weight loss (LWL, n = 5) cows, based on the percentage of BW loss between week 1 and 5 postpartum, were analyzed by proteomic analysis and bioinformatics (Ingenuity).
(TIF)

S2 Fig Protein expression of CB1 and FAAH in ATs collected at 14 d prepartum and 4 d PP from HWL and LWL cows. Cows were categorized as high-weight loss (HWL, n = 9) or low-weight loss (LWL, n = 9) based on the percentage of BW loss between week 1 and 5 postpartum. The protein abundances of CB1 (**A**) and FAAH (**B**) were assessed by Western blotting analysis and corrected by β-actin as an internal standard. Samples for the western blot were prepared (×3.5), divided, and loaded to separate gels that ran simultaneously. The CB1, FAAH, and β-actin ran in parallel gels due to the proximity of the bands. Data represent the mean ± SEM.
(TIF)

S3 Fig Uncropped immunoblots of PLIN, Actin, MGLL, CB1, and FAAH in ATs collected at 14 d prepartum and 4 d PP from HWL and LWL cows. Cows were categorized as high-weight loss (HWL, n = 9) or low-weight loss (LWL, n = 9) based on the percentage of BW loss between week 1 and 5 postpartum.
(TIF)

## References

[1] Drackley JK. ADSA Foundation Scholar Award. Biology of dairy cows during the transition period: the final frontier? J Dairy Sci. 1999;82: 2259–73. Available: http://www.ncbi.nlm.nih.gov/pubmed/10575597 10575597 10.3168/jds.s0022-0302(99)75474-3

[2] Bell AW, Bauman DE. Adaptations of glucose metabolism during pregnancy and lactation. J Mammary Gland Biol Neoplasia. 1997;2: 265–78. Available: http://www.ncbi.nlm.nih.gov/pubmed/10882310 10882310 10.1023/a:1026336505343

[3] Chilliard Y, Ferlay A, Faulconnier Y, Bonnet M, Rouel J, Bocquier F. Adipose tissue metabolism and its role in adaptations to undernutrition in ruminants. Proc Nutr Soc. 2000;59: 127–34. Available: http://www.ncbi.nlm.nih.gov/pubmed/10828182 10828182 10.1017/s002966510000015x

[4] Hammon HM, Stürmer G, Schneider F, Tuchscherer A, Blum H, Engelhard T, et al Performance and metabolic and endocrine changes with emphasis on glucose metabolism in high-yielding dairy cows with high and low fat content in liver after calving. J Dairy Sci. 2009;92: 1554–66. 10.3168/jds.2008-1634 19307636

[5] Zachut M, Honig H, Striem S, Zick Y, Boura-Halfon S, Moallem U. Periparturient dairy cows do not exhibit hepatic insulin resistance, yet adipose-specific insulin resistance occurs in cows prone to high weight loss. J Dairy Sci. American Dairy Science Association; 2013;96: 5656–69. Available: http://www.ncbi.nlm.nih.gov/pubmed/23871373 10.3168/jds.2012-6142 23871373

[6] Zachut M. Defining the adipose tissue proteome of dairy cows to reveal biomarkers related to peripartum insulin resistance and metabolic status. J Proteome Res. 2015;14: 2863–71. 10.1021/acs.jproteome.5b00190 26062109

[7] Zachut M, Moallem U. Consistent magnitude of postpartum body weight loss within cows across lactations and the relation to reproductive performance. J Dairy Sci. 2017;100 10.3168/jds.2016-11750 28131583

[8] Cable JC, Tan GD, Alexander SP, O Sullivan SE. The effects of obesity, diabetes and metabolic syndrome on the hydrolytic enzymes of the endocannabinoid system in animal and human adipocytes. Lipids Health Dis. 2014;13: 43 10.1186/1476-511X-13-43 24593280 PMC3995979

[9] Silvestri C, Ligresti A, Di Marzo V. Peripheral effects of the endocannabinoid system in energy homeostasis: adipose tissue, liver and skeletal muscle. Rev Endocr Metab Disord. 2011;12: 153–62. 10.1007/s11154-011-9167-3 21336842

[10] Di Marzo V, Piscitelli F, Mechoulam R. Cannabinoids and endocannabinoids in metabolic disorders with focus on diabetes. Handb Exp Pharmacol. 2011; 75–104. 10.1007/978-3-642-17214-4_4 21484568

[11] Lipina C, Rastedt W, Irving AJ, Hundal HS. New vistas for treatment of obesity and diabetes? Endocannabinoid signalling and metabolism in the modulation of energy balance. Bioessays. 2012;34: 681–91. 10.1002/bies.201200031 22674489

[12] Tam J, Hinden L, Drori A, Udi S, Azar S, Baraghithy S. The therapeutic potential of targeting the peripheral endocannabinoid/CB1 receptor system. Eur J Intern Med. 2018;49: 23–29. 10.1016/j.ejim.2018.01.009 29336868

[13] Lau BK, Cota D, Cristino L, Borgland SL. Endocannabinoid modulation of homeostatic and non-homeostatic feeding circuits. Neuropharmacology. 2017;124: 38–51. 10.1016/j.neuropharm.2017.05.033 28579186

[14] Silvestri C, Di Marzo V. The endocannabinoid system in energy homeostasis and the etiopathology of metabolic disorders. Cell Metab. 2013;17: 475–490. 10.1016/j.cmet.2013.03.001 23562074

[15] Devane WA, Hanus L, Breuer A, Pertwee RG, Stevenson LA, Griffin G, et al Isolation and structure of a brain constituent that binds to the cannabinoid receptor. Science. 1992;258: 1946–9. Available: http://www.ncbi.nlm.nih.gov/pubmed/1470919 1470919 10.1126/science.1470919

[16] Mechoulam R, Ben-Shabat S, Hanus L, Ligumsky M, Kaminski NE, Schatz AR, et al Identification of an endogenous 2-monoglyceride, present in canine gut, that binds to cannabinoid receptors. Biochem Pharmacol. 1995;50: 83–90. Available: http://www.ncbi.nlm.nih.gov/pubmed/7605349 7605349 10.1016/0006-2952(95)00109-d

[17] Sugiura T, Kondo S, Sukagawa A, Nakane S, Shinoda A, Itoh K, et al 2-Arachidonoylglycerol: a possible endogenous cannabinoid receptor ligand in brain. Biochem Biophys Res Commun. 1995;215: 89–97. Available: http://www.ncbi.nlm.nih.gov/pubmed/7575630 7575630 10.1006/bbrc.1995.2437

[18] Cristino L, Becker T, Di Marzo V. Endocannabinoids and energy homeostasis: An update. BioFactors. 2014;40: 389–397. 10.1002/biof.1168 24752980

[19] Kano M, Ohno-Shosaku T, Hashimotodani Y, Uchigashima M, Watanabe M. Endocannabinoid-mediated control of synaptic transmission. Physiol Rev. 2009;89: 309–80. 10.1152/physrev.00019.2008 19126760

[20] Jo Y-H, Chen Y-JJ, Chua SC, Talmage DA, Role LW. Integration of endocannabinoid and leptin signaling in an appetite-related neural circuit. Neuron. 2005;48: 1055–66. 10.1016/j.neuron.2005.10.021 16364907 PMC2280039

[21] Howlett AC. Pharmacology of cannabinoid receptors. Annu Rev Pharmacol Toxicol. 1995;35: 607–634. 10.1146/annurev.pa.35.040195.003135 7598509

[22] Pertwee RG, Ross RA. Cannabinoid receptors and their ligands. Prostaglandins, Leukot Essent Fat Acids. 2002;66: 101–121. 10.1054/plef.2001.0341 12052030

[23] Cota D, Marsicano G, Tschöp M, Grübler Y, Flachskamm C, Schubert M, et al The endogenous cannabinoid system affects energy balance via central orexigenic drive and peripheral lipogenesis. J Clin Invest. 2003;112: 423–31. 10.1172/JCI17725 12897210 PMC166293

[24] Bensaid M, Gary-Bobo M, Esclangon A, Maffrand JP, Le Fur G, Oury-Donat F, et al The cannabinoid CB1 receptor antagonist SR141716 increases Acrp30 mRNA expression in adipose tissue of obese fa/fa rats and in cultured adipocyte cells. Mol Pharmacol. 2003;63: 908–14. Available: http://www.ncbi.nlm.nih.gov/pubmed/12644592 12644592 10.1124/mol.63.4.908

[25] Roche R, Hoareau L, Bes-Houtmann S, Gonthier M-P, Laborde C, Baron J-F, et al Presence of the cannabinoid receptors, CB1 and CB2, in human omental and subcutaneous adipocytes. Histochem Cell Biol. 2006;126: 177–87. 10.1007/s00418-005-0127-4 16395612

[26] Blüher M, Engeli S, Klöting N, Berndt J, Fasshauer M, Bátkai S, et al Dysregulation of the peripheral and adipose tissue endocannabinoid system in human abdominal obesity. Diabetes. 2006;55: 3053–60. 10.2337/db06-0812 17065342 PMC2228260

[27] Matias I, Gonthier M-P, Orlando P, Martiadis V, De Petrocellis L, Cervino C, et al Regulation, function, and dysregulation of endocannabinoids in models of adipose and β-pancreatic cells and in obesity and hyperglycemia. J Clin Endocrinol Metab. 2006;91: 3171–3180. 10.1210/jc.2005-2679 16684820

[28] Khan MJ, Graugnard DE, Loor JJ. Endocannabinoid system and proopiomelanocortin gene expression in peripartal bovine liver in response to prepartal plane of nutrition. J Anim Physiol Anim Nutr (Berl). 2012;96: 907–919. 10.1111/j.1439-0396.2011.01204.x 21812840

[29] Zachut M, Kra G, Livshitz L, Portnick Y, Yakoby S, Friedlander G, et al Seasonal heat stress affects adipose tissue proteome toward enrichment of the Nrf2-mediated oxidative stress response in late-pregnant dairy cows. J Proteomics. 2017;158 10.1016/j.jprot.2017.02.011 28238905

[30] Honig H, Miron J, Lehrer H, Jackoby S, Zachut M, Zinou a, et al Performance and welfare of high-yielding dairy cows subjected to 5 or 8 cooling sessions daily under hot and humid climate. J Dairy Sci. 2012;95: 3736–42. Available: http://www.ncbi.nlm.nih.gov/pubmed/22720930 10.3168/jds.2011-5054 22720930

[31] AOAC. Official Methods of Analysis Arlington, VA: Association of Official Analytical Chemists; 1990.

[32] Van Soest PJ, Robertson JB, Lewis BA. Methods for Dietary Fiber, Neutral detergent fiber, and nonstarch polysaccharides in relation to animal nutrition. J Dairy Sci. 1991;74: 3583–3597. 10.3168/jds.S0022-0302(91)78551-2 1660498

[33] Feldman E.. Animal models of diabetic complications consortium (AMDCC protocols),. 2004.

[34] Knani I, Earley BJ, Udi S, Nemirovski A, Hadar R, Gammal A, et al Targeting the endocannabinoid/CB1 receptor system for treating obesity in Prader-Willi syndrome. Mol Metab. 2016;5: 1187–1199. 10.1016/j.molmet.2016.10.004 27900261 PMC5123200

[35] Shalit T, Elinger D, Savidor A, Gabashvili A, Levin Y. MS1-based label-free proteomics using a quadrupole orbitrap mass spectrometer. J Proteome Res. 2015;14: 1979–86. 10.1021/pr501045t 25780947

[36] Feise RJ. Do multiple outcome measures require p-value adjustment? BMC Med Res Methodol. 2002;2: 8 Available: http://www.ncbi.nlm.nih.gov/pubmed/12069695 10.1186/1471-2288-2-8 12069695 PMC117123

[37] Bradford BJ, Yuan K, Farney JK, Mamedova LK, Carpenter AJ. Invited review: Inflammation during the transition to lactation: New adventures with an old flame. J Dairy Sci. 2015;98: 6631–50. 10.3168/jds.2015-9683 26210279

[38] Oliveros JC. An interactive tool for comparing lists with Venn’s diagrams. Title. In: http://bioinfogp.cnb.csic.es/tools/venny/index.htm.

[39] Di Marzo V, Verrijken A, Hakkarainen A, Petrosino S, Mertens I, Lundbom N, et al Role of insulin as a negative regulator of plasma endocannabinoid levels in obese and nonobese subjects. Eur J Endocrinol. 2009;161: 715–722. 10.1530/EJE-09-0643 19745037

[40] Lo Verme J, Gaetani S, Fu J, Oveisi F, Burton K, Piomelli D. Regulation of food intake by oleoylethanolamide. Cell Mol Life Sci. 2005;62: 708–16. 10.1007/s00018-004-4494-0 15770421 PMC11924464

[41] Annuzzi G, Piscitelli F, Di Marino L, Patti L, Giacco R, Costabile G, et al Differential alterations of the concentrations of endocannabinoids and related lipids in the subcutaneous adipose tissue of obese diabetic patients. Lipids Health Dis. 2010;9: 43 10.1186/1476-511X-9-43 20426869 PMC2868848

[42] Osei-Hyiaman D, DePetrillo M, Pacher P, Liu J, Radaeva S, Bátkai S, et al Endocannabinoid activation at hepatic CB1 receptors stimulates fatty acid synthesis and contributes to diet-induced obesity. J Clin Invest. 2005;115: 1298–1305. 10.1172/JCI23057 15864349 PMC1087161

[43] Jamshidi N, Taylor DA. Anandamide administration into the ventromedial hypothalamus stimulates appetite in rats. Br J Pharmacol. 2001;134: 1151–4. 10.1038/sj.bjp.0704379 11704633 PMC1573067

[44] Cota D. Role of the Endocannabinoid System in Energy Balance Regulation and Obesity Obesity and Metabolism. Basel: KARGER; 2008 pp. 135–145. 10.1159/000115362 18230900

[45] Rodríguez de Fonseca F, Navarro M, Gómez R, Escuredo L, Nava F, Fu J, et al An anorexic lipid mediator regulated by feeding. Nature. 2001;414: 209–212. 10.1038/35102582 11700558

[46] Bennetzen MF, Wellner N, Ahmed SS, Ahmed SM, Diep TA, Hansen HS, et al Investigations of the human endocannabinoid system in two subcutaneous adipose tissue depots in lean subjects and in obese subjects before and after weight loss. Int J Obes. 2011;35: 1377–1384. 10.1038/ijo.2011.8 21326208

[47] Izzo AA, Piscitelli F, Capasso R, Aviello G, Romano B, Borrelli F, et al Peripheral endocannabinoid dysregulation in obesity: relation to intestinal motility and energy processing induced by food deprivation and re-feeding. Br J Pharmacol. 2009;158: 451–461. 10.1111/j.1476-5381.2009.00183.x 19371345 PMC2757684

[48] Starowicz KM, Cristino L, Matias I, Capasso R, Racioppi A, Izzo AA, et al Endocannabinoid dysregulation in the pancreas and adipose tissue of mice fed with a high-fat diet. Obesity. 2008;16: 553–565. 10.1038/oby.2007.106 18239598

[49] Sidibeh CO, Pereira MJ, Lau Börjesson J, Kamble PG, Skrtic S, Katsogiannos P, et al Role of cannabinoid receptor 1 in human adipose tissue for lipolysis regulation and insulin resistance. Endocrine. 2017;55: 839–852. 10.1007/s12020-016-1172-6 27858284 PMC5316391

[50] Pagano C, Pilon C, Calcagno A, Urbanet R, Rossato M, Milan G, et al The endogenous cannabinoid system stimulates glucose uptake in human fat cells via phosphatidylinositol 3-kinase and calcium-dependent mechanisms. J Clin Endocrinol Metab. 2007;92: 4810–9. 10.1210/jc.2007-0768 17785353

[51] Deveaux V, Cadoudal T, Ichigotani Y, Teixeira-Clerc F, Louvet A, Manin S, et al Cannabinoid CB2 Receptor Potentiates Obesity-Associated Inflammation, Insulin Resistance and Hepatic Steatosis. Polidori C, editor. PLoS One. 2009;4: e5844 10.1371/journal.pone.0005844 19513120 PMC2688760

[52] Mallat A, Lotersztajn S. Cannabinoid receptors as therapeutic targets in the management of liver diseases. Drug News Perspect. 2008;21: 363 10.1358/dnp.2008.21.7.1255306 19259549

[53] Contreras GA, Strieder-Barboza C, De Koster J. Symposium review: Modulating adipose tissue lipolysis and remodeling to improve immune function during the transition period and early lactation of dairy cows. J Dairy Sci. 2018;101: 2737–2752. 10.3168/jds.2017-13340 29102145

[54] Bonsale R, Seyed Sharifi R, Dirandeh E, Hedayat N, Mojtahedin A, Ghorbanalinia M, et al Endocannabinoids as endometrial inflammatory markers in lactating Holstein cows. Reprod Domest Anim. 2018;53: 769–775. 10.1111/rda.13169 29542183

[55] Ceciliani F, Pocacqua V. The acute phase protein alpha1-acid glycoprotein: a model for altered glycosylation during diseases. Curr Protein Pept Sci. 2007;8: 91–108. Available: http://www.ncbi.nlm.nih.gov/pubmed/17305563 17305563 10.2174/138920307779941497

[56] Lykkesfeldt J, Svendsen O. Oxidants and antioxidants in disease: Oxidative stress in farm animals. Vet J. 2007;173: 502–511. 10.1016/j.tvjl.2006.06.005 16914330

[57] Bernabucci U, Ronchi B, Lacetera N, Nardone A. Influence of body condition score on relationships between metabolic satus and oxidative stress in periparturient dairy cows. J Dairy Sci. 2005;88: 2017–2026. 10.3168/jds.S0022-0302(05)72878-2 15905432

[58] Weems YS, Lewis AW, Neuendorff DA, Randel RD, Weems CW. Endocannabinoid 1 and 2 (CB(1); CB(2)) receptor agonists affect negatively cow luteal function in vitro. Prostaglandins Other Lipid Mediat. 2009;90: 89–93. 10.1016/j.prostaglandins.2009.09.003 19765667

[59] Tsutahara NM, Weems YS, Arreguin-Arevalo JA, Nett TM, LaPorte ME, Uchida J, et al Effects of endocannabinoid 1 and 2 (CB1; CB2) receptor agonists on luteal weight, circulating progesterone, luteal mRNA for luteinizing hormone (LH) receptors, and luteal unoccupied and occupied receptors for LH in vivo in ewes. Prostaglandins Other Lipid Mediat. 2011;94: 17–24. 10.1016/j.prostaglandins.2010.11.002 21109016

