# Supplementary figures and images for: Characterization of the endocannabinoid system in subcutaneous adipose tissue in periparturient dairy cows and its association to metabolic profiles
Source: PLoS One. 2018 Nov 7;13(11):e0205996. doi: 10.1371/journal.pone.0205996 (PMC6221292; doi:10.1371/journal.pone.0205996)

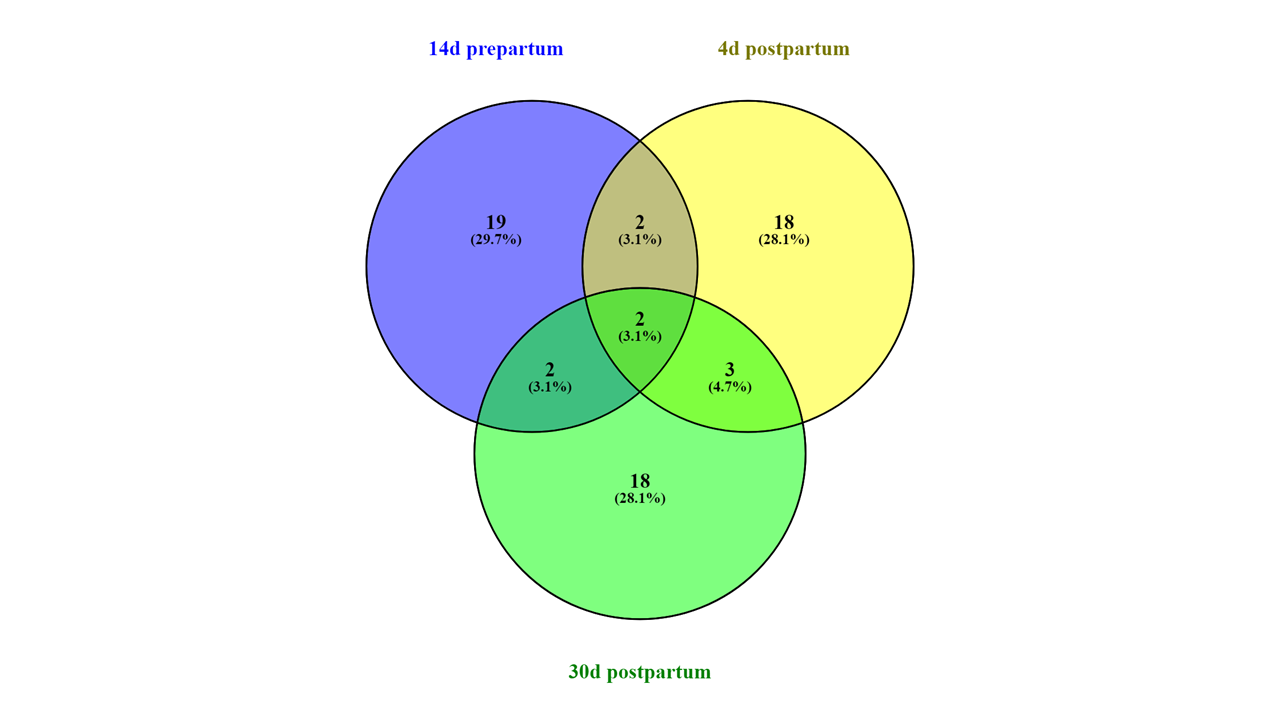

Supplement: S1 Fig — AT samples from high-weight loss (HWL, n = 5) or low-weight loss (LWL, n = 5) cows, based on the percentage of BW loss between week 1 and 5 postpartum, were analyzed by proteomic analysis and bioinformatics (Ingenuity). (TIF) [file pone.0205996.s002.tif]

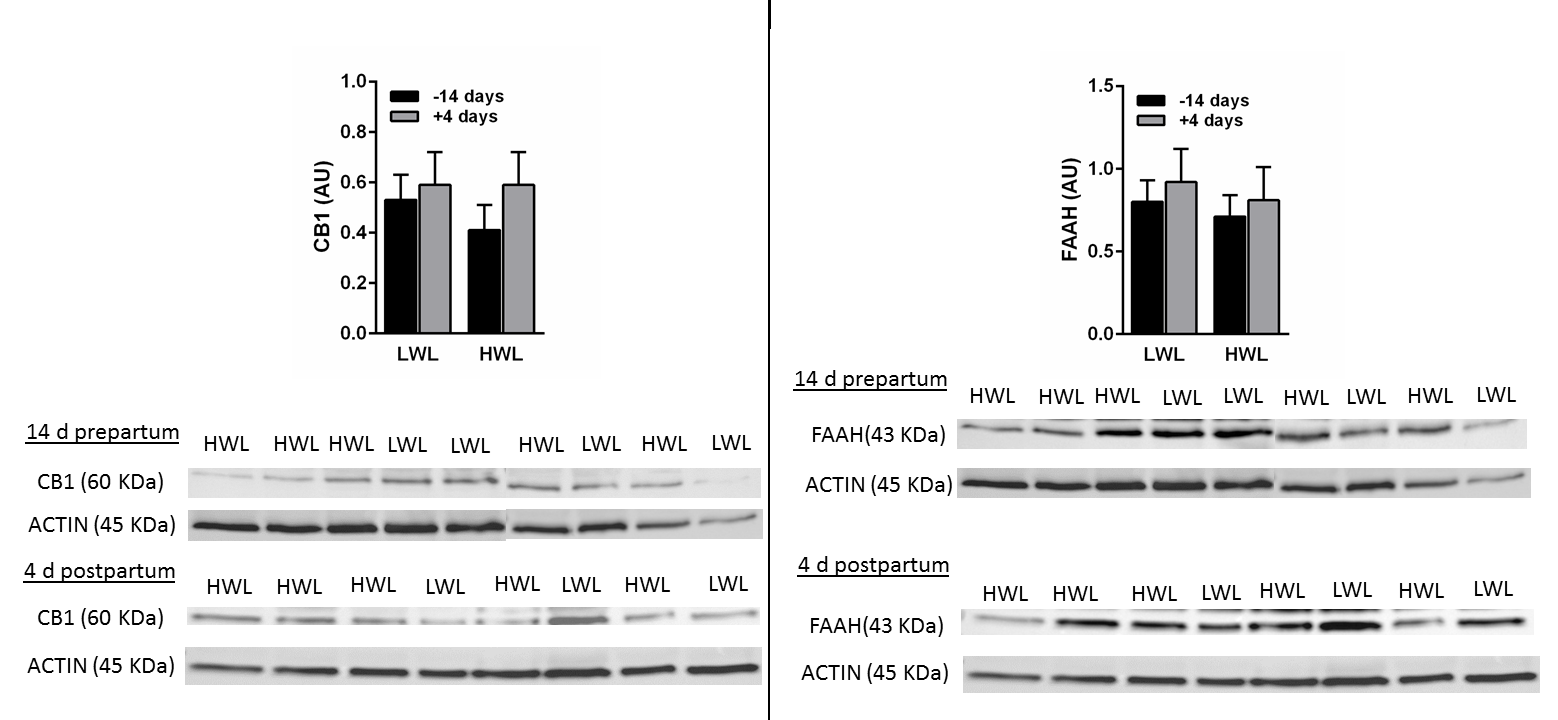

Supplement: S2 Fig — Cows were categorized as high-weight loss (HWL, n = 9) or low-weight loss (LWL, n = 9) based on the percentage of BW loss between week 1 and 5 postpartum. The protein abundances of CB1 (A) and FAAH (B) were assessed by Western blotting analysis and corrected by β-actin as an internal standard. Samples for the western blot were prepared (×3.5), divided, and loaded to separate gels that ran simultaneously. The CB1, FAAH, and β-actin ran in parallel gels due to the proximity of the bands. Data represent the mean ± SEM. (TIF) [file pone.0205996.s003.tif]

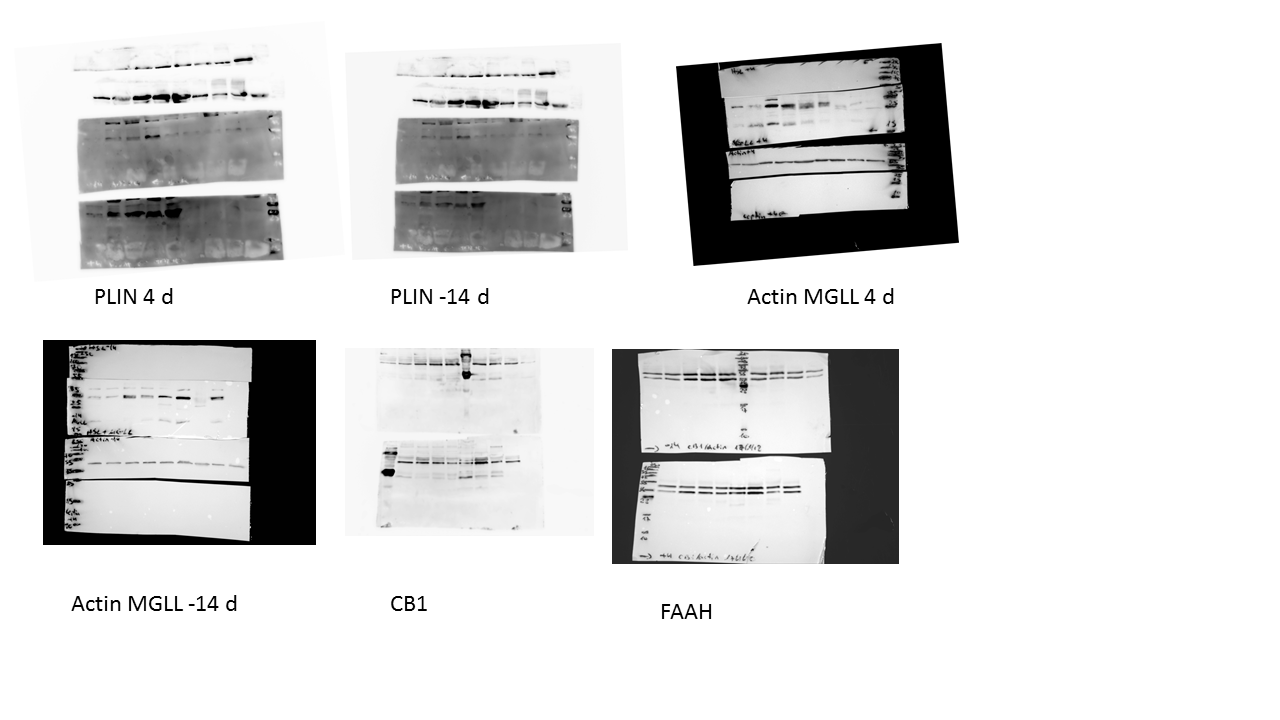

Supplement: S3 Fig — Cows were categorized as high-weight loss (HWL, n = 9) or low-weight loss (LWL, n = 9) based on the percentage of BW loss between week 1 and 5 postpartum. (TIF) [file pone.0205996.s004.tif]
